# Book Reviews

**Published:** 1809-02

**Authors:** 


### CRITICAL ANALYSIS
### OF THE
### RECENT PUBLICATIONS
### ON THE
### DIFFERENT BRANCHES OF PHYSIC, SURGERY, AND MEDICAL PHILOSOPHY.

---

### *The Edinburgh Medical and Surgical Journal, No. XVII.*

ARTICLE 1.—*Practical Observations on the Mode of making the Incision of the Cornea, for the Extraction of the Cataract.* By JAMES WARDROP, Fellow of the Royal Society, and of the Royal College of Surgeons of Edinburgh.

THIS is a very useful paper on a delicate operation. Its design is to introduce an instrument (invented by Mr. Beer, an occulist at Vienna) for cutting the cornea in the operation for extracting the cataract. The great advantage proposed, is to avoid cutting the cornea in such a way as to leave the iris entirely undefended in the lower part. The manner in which this is accomplished is very accurately described by Mr. Wardrop. As it is impossible to do justice to the paper by any means of abbreviation, we shall only recommend the careful perusal of it to such as engage in this delicate operation.

ARTICLE 2.—*Observations on the Utility of Venesection in Purpura.* By C. H. PARRY, Physician, Bath.

As we feel personally concerned in this paper, we shall, as on all such occasions, transcribe the author's words, with *as much of the context as is necessary to show his full meaning.*

"In the remarks on the last part of Dr. Willan's Essay on Cutaneous Diseases, contained in the 115th number of the London Medical and Physical Journal, speaking of sea-scurvy, the writer observes, that all the cases of purpura 'have so near an affinity with it, that if they are really different, we wish the author had accurately marked the distinction.' After which, he quotes Dr. Willan himself, as saying, 'cases of purpura seem to have been studiously multiplied in periodical publications, and in medical and chirurgical miscellanies. I consider it, under all the forms described, as pertaining to scurvy.' These, I presume, are the popular opinions on the subject. Whether they are universally just, may be learned from the following cases."

We shall now transcribe our own words, as they are contained in a different volume.

"The fifth division of this order, is *Purpura,* by which term the author 'proposes, with Riverius and some others, to express an efflorescence, consisting of small, distinct, purple specks and patches, attended with general debility, but not always with fever.

Three

Three striking varieties of the complaint, thus defined, may be denominated Purpura simplex, Purpura hæmorrhagica, and Purpura urticans, no one of which is contagious.'—All these, particularly the second, have so near an affinity with stomacace, or sea-scurvy, that if they are really different, we wish the author had accurately marked the distinction. It is unnecessary for us to add, that sea-scurvy is now no longer imputed to the mere salt in salted provisions, but to 'a sedentary mode of life, impure air, anxiety of mind, want of proper food.' We should have been more cautious in this remark, on so judicious a writer, did not the nature of our work afford him an opportunity of giving us, and a large portion of his readers, that information which we shall be happy to receive from such a source."

The reader will see by this, that we express nothing like a doubt, that purpura may exist without the other symptoms of sea-scurvy, but only our wish, if Dr. Willan had referred to any other, that he would have expressed himself more pointedly. All Dr. Willan's divisions are described as attended with debility, and the remedy is bark with muriated acid or muriated tincture of iron. Dr. Parry's are quite different; we shall transcribe one of them as a specimen.

"Ten or twelve years ago I was sent for to attend a lady about 50 years of age, of a fair complexion, and rather full habit, who, for some days, had laboured under slight febrile symptoms, with a full pulse, though but little thirst or fur on her tongue. Nothing else was worthy of attention but the state of her skin, which was thickly sprinkled with spots, small, and of irregular forms, not raised above the surface, of a dark logwood colour, and in no degree evanescent on pressure. An apothecary in this city, Mr. Foster, had, previously to my visit, taken from her arm at least fourteen ounces of blood, which I saw in a bason, and the surface of which consisted of a crust of coagulated lymph, as thick and tenacious as I ever witnessed in the most acute case of rheumatism, pleurisy, or hepatitis. The cruor was also very firm and cohesive, and difficult of diffusion when shaken in the serum; notwithstanding which, the proportion of the whole crassamentum to that of the serum was uncommonly great. The patient expressed so much relief from the bleeding, that I thought myself justified in ordering it to be shortly afterwards repeated. Relief was by this measure again obtained; and, under the use of some common refrigerants, the lady quickly recovered, without bark, or any other of those remedies usually dignified with the name of tonics."

No one can mistake these or any of the subsequent cases with the mode of treatment as related by Dr. Parry, for sea-scurvy. We consider the public much obliged by the communication, and are well pleased if our remark has produced it. We are glad to find too that Dr. Parry is collecting other facts on this subject, which he means to lay before the public. The present paper concludes with the following judicious remarks.

"In the mean while, whatever may be the nature of sea-scurvy, or or of purpura in general, of which every experienced medical practitioner must have seen numerous examples, there can be little doubt, that the cases which I have related are to be considered as of the nature of what are called active hæmorrhages; since it matters not, in a pathological view, whether febrile extravasation of blood takes place from the rupture, or gaping of an artery in the cellular membrane, in the skin, or on the surface of the epithelion, in the nose, fauces, or bronchia."

Article 3.—*Observations on an Instance of Condensed Lungs; published in the Edinburgh Medical Journal, Vol. IV. p.* 301. By another Physician.

The paper to which these observations allude, has been already noticed by us, see vol. xx, p. 178. The remarks of this *other physician* serve to confirm the opinion we formed of the case.

Article 4.—*Experiments, shewing that a Mineral Poison may produce sudden and violent Death, and yet may be incapable of Detection in the Contents of the Stomach.* By John Bostock, M. D. Liverpool; in a Letter to Dr. Duncan, Jun.

These experiments were made in consequence of a controversy in Liverpool, which we have left unnoticed till we can produce an impartial statement of the whole. They are conducted with that accuracy which might be expected of Dr. Bostock. The following is the result of the whole.

"1. The fluid taken from the dog's stomach contained muriatic acid, probably in the form of common salt, and animal matter, probably mucus, in considerable quantity.

"2. The tests that were employed to discover the oxy-muriate of mercury, were capable of detecting it in a fluid, when it composed only $\frac{1}{3000000}$ of its weight.

"3. These tests did not detect any of the oxy-muriate of mercury in the fluid taken from the dog's stomach; it may therefore be concluded,

"4. That an animal may be suddenly killed by receiving a metallic poison into the stomach, and yet that the nicest tests may not be able to detect any portion of the poison, after death, in the contents of the stomach.

"This conclusion appears incontrovertible; and though some analogous facts had occasionally been noticed, it is so different from the generally received opinion upon the subject, that I think it must have considerable influence on all future judicial proceedings, in which the question of poisoning is agitated.

"*Liverpool, Nov.* 19, 1808."

Article 5.—*On the Origin of Calculi.* By James Barlow, Esq. Surgeon, Blackburn, Lancashire.

The object of this is to show, as was many years ago suggested by Dr. Austin, that urinary and other calculi are formed more from mucus than from any deposition of the urine in the bladder, or of the contents of any other cavity. At the conclusion of the paper, paper, a doubt is suggested whether some of those calculi which are found in young subjects, who, after the operation, show no disposition to form new concretions, have not their commencement before birth. This opinion is supported by the sabulous matter found deposited in the bladder of some fœtus.

ARTICLE 6.—*Observations on the Use of Arsenic.* By G. N. HILL, Surgeon, Chester.

BESIDES intermittents, the author recommends the use of arsenic in a variety of chronic diseases. The present paper comprehends a general state of anorexia, which sometimes ends in typhus fever, chronic ophthalmia, and paralysis. In all tedious complaints it is no inconsiderable advantage to have a remedy at hand, which may be given in any form, yet need never be disgusting to the palate.

ARTICLE 7.—*Biographical Notices of* Dr. OSBORNE *and* Dr. LUBBOCK.

THESE anecdotes comprehend nothing more of these respectable physicians than is generally known.

ARTICLE 8.—*Some Account of Cretinism.* By HENRY REEVE, M.D. Norwich. Communicated by the Author.

THIS judicious paper is similar to the one presented by the same gentleman to the Royal Society.

ARTICLE 9.—*Report on a Memoir of Drs. Gall and Spurzheim, relative to the Anatomy of the Brain.* By M. M. TENON, PORTAL, SABATIER, PINEL, and CUVIER, presented to, and adopted by the Class of Mathematical and Physical Sciences of the National Institute.

THOSE who are acquainted with the prolixity of French reports, whether academic or judicial, will gladly excuse our giving the whole paper before us. We have with much industry selected the outlines, preserving the most important facts.

We are first to remark, that these observations relate principally to the anatomy of the brain, and some deductions immediately connected with parts and particularities of structure discovered by the authors. These theories, which have so long been talked of, and which must have been imperfectly explained, as we derive them principally from his disciples, without the sanction of Dr. Gall, have no necessary reference to the subject of this paper.

" Forgetting, therefore, (says the Report) entirely what has been said and written for and against Dr. Gall, in public, in Journals, or in pamphlets, not even confining ourselves solely to his memoir, which has not appeared to us to be written with all the order and perspicuity to be desired, we invited him, as well as Dr. Spurzheim, to our conferences. They readily dissected the brain in our presence, and we dissected it before them ; we afterwards re-examined, by ourselves, the observations they communicated to us ; lastly, we endeavoured for a time to adopt their manner of viewing the subject, and to give a clear and precise abstract of it, which we submitted submitted to them, that they might be satisfied whether we had rightly comprehended their ideas.

"After taking all these precautions, we endeavoured to ascertain what was new or just in their ideas, and in the consequences which the authors deduced from them."

After this, follows some remarks, more metaphysical than physiological, before the mode of dissection pursued by Dr. Gall and the reporters, is described. By this mode, first sketched by Varolius, the under part of the brain is first examined, and the *medulla oblongata* is followed across the *pons Varolii*, the *thalami optici*, and *corpora striata;* its fibres are seen expanding to form the hemispheres; it is even possible to stretch out the hemispheres by removing their lateral attachment to the crura cerebri; to divide longitudinally the spinal marrow and the cerebellum; and then each half of the former is seen forming a pedicle, which is implanted into the side of the hemisphere, as the stem of a mushroom is fixed into its *pileus*.

This mode possesses the very great advantage of allowing us to follow more easily the direction of the medullary fibres, the only circumstance which can afford us any idea with regard to the course of the cerebral functions; and it is probable, that it would have been more generally adopted, if Varolius had not represented it in a very coarse figure, and if the work of Vieussens had not always remained, for what reason it is difficult to determine, in a kind of discredit which it did not deserve.

It is nearly this method of Varolius, which is followed by Drs. Gall and Spurzheim, and which a part of their memoir is intended to defend; a labour, indeed, very useless, for so complicated an organ as the brain ought to be examined on every side; it must be cut into in every way; and whenever any plan is discovered which points out some new circumstance, it furnishes an important addition to anatomy.

So little is known of the functions of the brain, and so little are philosophers willing to offer conjectures on a subject they cannot comprehend, that most have satisfied themselves with a description of the parts, and some general inferences, that the nerves are processes from the brain; that the cortical substance of the hemispheres and of the cerebellum being nearly altogether vascular, forms a secreting organ, and the medullary substance is a collection of excreting vessels, or conducting filaments. That the medulla oblongata and spinal marrow are a bundle larger than the others, from which the different pairs of spinal nerves go off in succession. That this, the inference of the nervous system on organic life, as well as all the efforts of the will, descend from the brain along the nerves. But with all who have the courage to speak out, a difficulty remains concerning the centre at which these combinations, or in other words, what shall be made the seat of the soul.

There would be no difficulty in shewing the inconclusiveness of these opinions, not only by insects, who, after a division of the supposed supposed seat of the soul, can still support an existence, and perform many of their customary functions; but by those human monsters, which in the fœtal state, have grown to maturity without any brain, and with all the nerves in the rest of the body. Mr. Hunter, too cautious to attempt explaining what probably never can be explained, was, however, anxious to show that the only use hitherto discovered for brain and nerves, was to preserve a communication of sensation and action throughout the whole body. Hence it followed, that the part might retain its own sensation and its own action, when the nervous communication was destroyed, yet the means of preserving from external injury would be lost, inasmuch as the communication with the senses being destroyed, no sufficient provision could be made for its protection.

The opinions, therefore, of Drs. Gall and Spurzheim, that the nervous system is a net-work, will not be controverted, *in limine*, by those whose only wish is to be informed. We shall, therefore, proceed to consider their doctrine, concerning the *links and knots of which this net-work is composed.* These the Reporters have divided into the ten following heads.

" 1. The cineritious matter is the *matrix of the medullary filaments;* wherever it exists, these filaments are produced; it exists wherever they are produced. Whenever a medullary bundle crosses cineritious matter, it is enlarged by filaments received from the latter, and none of these bundles enlarge without the concurrence of this matter, whether it form a sensible enlargement, or only follow and accompany the medullary bundle.

" 2. The spinal marrow is not a bundle of nerves descending from the brain. The spinal nerves arise by filaments, some of which ascend, others descend; this is observed especially in brute animals. The cineritious matter of the internal part of the spinal marrow is the matrix of these filaments; the spinal marrow swells wherever it gives off a pair of nerves, and the more the larger that these nerves are. Thus the spinal marrow of the larger animals, as well as that of insects and worms with red blood, is only a series of ganglia, which give off nerves; but all these ganglia communicate with each other.

" 3. The nerves commonly called cerebral, and which arise from the under part of the encephalon, and chiefly from the medulla oblongata, do not come from the brain any more than the others; on the contrary, when we follow separately the roots of each of these into the substance of the medulla oblongata, we see that they reascend from the medulla towards the point where they appear externally, and that they do not descend from the brain, to pass through the medulla oblongata.

" 4. The brain and cerebellum are themselves only developements of bundles which have come from the medulla oblongata, in the same manner as the nerves proceed from it.

" The brain, in particular, arises principally from the bundles called pyramidal eminences, which cross each other in passing out of of the medulla oblongata, each going to the opposite side to that from which it comes, they swell out first in crossing the *pons varolii*, again in passing over the tubercles called *thalami optici*, and a third time in those tubercles called *corpora striata*, always from the addition of medullary filaments, which the cineritious matter, contained in these three parts, adds to those they originally consisted of. The cerebellum comes from bundles called *processus cerebelli ad medullam*, or *corpora restiformia*, which receive an addition of filaments once only from the cineritious matter of what is called *corpus ciliare*.

"5. These two pairs of bundles, after receiving these additions, after having assumed consequently a diverging direction, terminate each in two large expansions, covered externally by cineritious matter, which only here deserves the name of *cortical* substance; and these expansions, folded in various ways, form what are called the hemispheres of the brain, the lobes, and the *vermiform processes* of the cerebellum.

"6. From the whole extent of these expansions, other medullary filaments are produced, which converge from the two sides of the brain and cerebellum, towards the middle line, where the filaments of the one side unite with those of the other, and form what are called the *commissures*.

"The corpus callosum, the fornix and its appendages, form the largest of the commissures of the brain; the anterior commissure is that which joins the middle lobes. The commissure of the cerebellum is composed of the transverse layers of the pons varolii.

"7. When we remove or lacerate the converging fibres which go to the corpus callosum, and which serve as a cieling to the lateral ventricles, there only remains, under the cineritious substance, a medullary part lining it, and following all its folds; and far from forming a solid mass, as has been hitherto supposed, there is always in the middle of each convolution of the brain and cerebellum a solution of continuity, and by employing proper care, we may unfold this portion of the medulla, as we would unfold the cineritious substance if it were alone. In a word, each convolution is a kind of small purse or canal, closed externally by a double layer of cineritious and medullary matter, and on the side of the ventricle by the converging medullary fibres.

"8. As the pairs of bundles forming the brain and cerebellum have their commissures, those which form the nerves have often theirs also, which may be very easily demonstrated in the second, the fourth, fifth, and seventh pairs, and very probably in the others.

"9. The ganglia spread over the whole body are small masses of cineritious matter, which certain nerves pass through, and where they are strengthened as the pedunculi of the brain are increased in the thalami optici and corpora striata.

"These two pairs of tubercles, then, are true ganglia to these pedunculi. The cineritious matter of the cortical part of the brain and cerebellum may likewise be regarded as a ganglion of the commissures, or converging fibres. That of the inner part of of the spinal marrow forms, in like manner, the primary ganglia of the spinal nerves. The cerebral nerves have probably each of them a particular ganglion, and it is easy to demonstrate it in several of them. The cineritious matter, and consequently the ganglia, may therefore be compared to the mucous expansion which covers all the extremities of the nerves of the skin, of the intestines, and even the pulp of the labyrinth, and the sort of mucous varnish which covers the retina.

" 10. From these nine articles, all merely anatomical, all more or less susceptible of being verified, a tenth results, which completes and is the essential character of the anatomical doctrine of Drs. Gall and Spurzheim, viz. that each pair of nerves forms a particular system; that all these systems communicate together, and re-unite in the great cord of the medulla oblongata and spinal marrow; and, lastly, that the brain and cerebellum, far from being the origin or source of this cord, are, on the contrary, an appendix or sort of *diverticulum*, reserved for certain functions, but receiving an influence from all the parts of the cord, and exercising one upon them by means of their communications."

The secreting property of the cineritious substance is rarely disputed as a probability much confirmed by the immense quantity of blood which passes through it, and passes so slowly.

The second article refers to the spinal marrow of insects and articulated worms, or those with red blood. In these it is well known that the brain is scarcely larger than the ganglions, or knots, as they are here called, which appear at those parts of the spinal marrow from which the pairs of nerves go off. Some of these insects, it is remarked, are actually divisible into perfect individuals, and though this cannot be accomplished in animals of larger brains, yet in man it is observed that the medulla swells wherever the nerves are given off; and in a preparation of the spinal marrow of the calf, a slight swelling was distinguishable between every pair of nerves.

For the sake of a more accurate examination, the 3d article is subdivided into as many heads as there are pairs of nerves, in order to prove that all the nerves arise from the medulla oblongata, and not from the brain. To give fair justice to the question we shall confine ourselves to a single organ.

" The optic nerve, say the Reporters, is very generally considered as proceeding from the thalami optici, because its roots are spread out upon them in the manner of a thin membranous expansion, which almost entirely covers them. However, anatomists have not been wanting, who supposed that they could trace at least a great part of these, as far as the nates; such as Morgagni, Winslow, Zinn. Santorini describes this origin very carefully, and adds another, which he makes to come from the testes; his pupil and editor, Girardi, confirms it. Vicq-d'Azyr, who was also very well acquainted with these connections of the optic nerves with the tubercula quadrigemina, pretends, however, that they have likewise other roots in the substance of the thalami, which, in the form of

 innumerable innumerable filaments, join the nerve in a great part of the course which it takes in surrounding the crus cerebri: and he boasted of this discovery. But this appears to us to be a deception, into which he may have been led by his mode of making parallel incisions. It is very true, that a number of white filaments arise from the cineritious substance of the thalami; but they do not appear to us to go to the optic nerve. On the contrary, they are added to the bundle which comes from the pyramidal eminences, as we shall mention presently. Drs. Gall and Spurzheim have found out a mode of dissection which demonstrates this very well, and which we shall mention again.

" They suppose then that it is possible, at least in several animals, to remove from above the thalami, without affecting them, the medullary expansion of the roots of the optic nerves, and to follow them as far as the internal part of the nates, where they form a white lamina which occupies the centre of these tubercles.

" The last point is certain; with respect to the first, as it can only be performed with the assistance of the handle of the scalpel, it is liable to the same doubt as all similar operations which may be tried on the brain.

" Our anatomists farther remark, that in those individuals in which the optic nerve of one side is weakened and thinner, the corresponding tubercle is also smaller; and that in those species which have large optic nerves, the nates are of larger size; but that frequently in these cases the thalami are smaller.

" The medullary tract which comes from the nates, meets in its course the tubercle called *corpus geniculatum externum.*

" That which comes from the *testes*, divides the first at the *corpus geniculatum internum*, and seems to slip under it, to cross it in passing forwards; thus Drs. Gall and Spurzheim do not believe that it belongs to the optic nerve; they have even for a long time thought that it forms the external root of the *olfactory*, which is indeed very nearly in that direction; but they have never been able to trace its continuity.

" One of our number has made several researches on this part of the brain, which appears to have been only well known to Santorini and Vicq-d'Azyr, but which has never been properly delineated.

" It is a fact, that in all quadrupeds, the principal bundle of the optic nerve goes from the nates to the *corpus geniculatum externum.*

" It is also certain, that another bundle comes from the testes, which makes an angle with the first; and which, after swelling out to form the *corpus geniculatum internum*, seems to pass under the first bundle, and proceed further, but is soon lost to the eye and the knife.

" *Lastly*, It is ascertained, that both the *testis* and the *corpus geniculatum internum*, are much larger in the carnivorous than in other animals; and that this fact would be favourable to the idea, that they contribute to form the olfactory nerve, which is so conspicuous in that class.

" But

"But we think that we have seen in the ape, that the *corpus geniculatum internum* receives a bundle from the *nates,* as well as from the *testes,* and forms by their re-union a root of the nerve, which joins only very low down that which comes as usual from the nates above the *thalamus opticus.* Drs. Gall and Spurzheim have themselves besides remarked, what one of us pointed out long ago, that the dolphins and porpoises, which have no olfactory nerve, have, however, very considerable *testes.*

"These animals, likewise, have the corpora striata as the others, which shows that these bodies do not possess the function attributed to them, of forming the olfactory nerve.

"The first pair, then, will be the only one, the roots of which cannot yet be traced to the medulla oblongata, and which does not agree as yet clearly with the rule established in the memoir which we are examining.

"Dr. Gall explains, conformably to the law mentioned in his first article, the enlargement of the optic nerves below their conjunction, by numerous filaments sent to them by the cineritious lamina interposed at the fore part of that conjunction; which filaments have been accurately described, and carefully delineated, by Vicq-d'Azyr.

"A strong objection was made to the origin, which our anatomists assign to the optic nerve, drawn from the structure of birds, which, it was said, have no *nates,* although their eye and optic nerve are of a very large size; but their answer is convincing. What Willis, Collins, Haller, and other anatomists have called thalami optici in birds, is in fact the *nates.* The true thalami optici are situated before, with their third ventricle, the pedicles of the pineal gland, and the two commissures in their usual place; in a word, they are in every respect similar to those of the quadrupeds, in proportion to their relative size; the supposed thalami of Haller are, on the contrary, between the posterior commissure and the valvula Vieussenii; the aqueduct of Sylvius passes between them; it is with it that the ventricles communicate, which are peculiar to them in this class.

"We have proved the truth of this important remark; it can admit of no reply. It is the more the duty of the Reporter to acknowledge it, as he had adopted the common error in his works.

"Now, as the tubercles in question evidently give rise to the optic nerves in birds, they confirm the origin which they ascribe to these nerves in the mammalia, and in man, instead of invalidating it.

"We may here repeat the elegant remark made by Vicq-d'Azyr, that these tubercles have a ventricle in birds in which the sense of sight is the more acute, as the olfactory nerves in the mammalia, in which the sense of smell is superior to the others."

In the Fourth Article, we have seen that Dr. Gall considers the brain and cerebellum as only developements of the bundles which have come from the medulla oblongata, in the same manner as the nerves nerves proceed from it. In explaining this connection between the medulla oblongata, the brain, and cerebellum, the professors require, besides the usual horizontal incisions made in examining the brain, a vertical incision, by which they undertake to explain how the medullary bundles enlarge, and to point out the true termination of the filaments of the thalamus opticus. This section passes through the middle of the pyramidal eminence of the crus cerebri, of the thalamus and corpus striatum on on side, obliquely forwards and outwards.

"The bundles of the pyramids are there distinctly seen intermixing with those of the pons varolii, and with the cineritious substance which mixes there with them, and supplies some additions; passing from thence into the crus cerebri, they again receive filaments from the *processus cerebelli ad testes.* At one place, under the thalamus opticus, they unite into a white mass, to which innumerable filaments of the internal part of the thalamus are joined at acute angles pointing forwards. It is essential to remark the latter circumstance; it proves that the thalami send their filaments forwards and not backwards, as Vieussens had supposed; it points out also, that it is not into the optic nerve that these filaments go, as Vicq-d'Azyr thought.

"The white mass then becomes stronger, and divides into a great number of diverging pillars, which constitute the white streaks of the middle of the corpora striata; the cineritious matter of the superior surface of these bodies gives also a great number of small filaments, as the thalami had given; at last, all these fibres are dispersed in the medullary mass of the hemispheres, where we shall follow them presently.

"The two transverse whitish arches, which are seen in the horizontal cut, and part of which Vicq-d'Azyr has expressed in his plate, are the places to which the greater number of filaments come from the superior parts of the thalami and corpora striata.

"Such is the faithful description of what is perceived by the eye; one of us has delineated all this arrangement in man, quadrupeds, and birds, in which the essential points are nearly the same.

"We are aware that there is no better reason for saying that the great fibrous bundles go from the pyramids to the hemispheres, than from the hemispheres to the pyramids; since the nervous influence proceeds in both directions.

"But one may and ought to inquire, which direction the small fibres of the thalami and corpora striati take. Are they supplied by these tubercles to enlarge the great medullary bundle, or are they given off from the medullary bundle to sink into these tubercles? This latter opinion certainly could have no probability, and no one will condemn Doctors Gall and Spurzheim for adopting the opposite opinion.

"They would then be in the right in this sense, when they say that that the medullary bundles go on always enlarging from the pyramids to the hemispheres.

"But whence do the inferior extremities of the bundles, these same pyramidal eminences, come? or whither do they go?

"They cross each other about two finger's breadth behind the pons varolii, and immediately disappear behind that point, losing themselves on both sides in the two cords composing the inferior surface of the spinal marrow.

"This is one of the most interesting facts in physiology and pathology.

"Every body knows how frequent it is to see a paralysis of one side, occasioned by an injury on the opposite side of the brain; physicians of every age have endeavoured to explain this fact by a decussation which they vaguely supposed in the fibres of the brain, or in the deepest roots of the nerves.

"However, we see, almost every where, only transverse fibres, commissures, and not crossed fibres.

"There is only one place, at the posterior extremity of the medulla oblongata, which presents a true decussation, which was discovered by Domenico Mistichelli, and very well described by him in 1709. Francois Pourfour du Petit likewise described it the following year, and was the first who made it known in France.

"How has it happened, that a point of structure so evident, adopted by Winslow, Lieutaud, Portal, distinctly described and plainly delineated by Santorini, should have been doubted by the great Haller, recently denied by very skilful men, and confounded by others, among whom may be reckoned Vicq-d'Azyr himself, with that of the transverse fibres which reunite, in their whole length, the lateral parts of the medulla oblongata?

"It is probably owing to the want of a sufficiently clear description, and perhaps also, because the place of the decussation must be often cut in separating the head from the body.

"It will be impossible to be mistaken in it, after the demonstrations of Drs. Gall and Spurzheim. When we separate from each other the two inferior cords of the medulla oblongata and spinal marrow, we see that they are separated by a pretty deep fissure, the bottom of which is occupied by transverse medullary filaments. This fissure is only interrupted at one place, which is what now engages our attention, and which is only two or three lines in length. The fibres of the pyramidal eminence of one side form there three or four filaments, which decussate upon the fissure with the opposite filaments, as the hairs of a mat, and which are blended afterwards with the rest of the medullary cord, into which they thus enter obliquely.

"This decussation strikes the eye when we separate gently the edges of the longitudinal fissure of the medulla oblongata, because it is the only place where we cannot perceive the bottom of the fissure.

"There is certainly some merit in having renewed the general

 knowledge knowledge of an important point of doctrine, which the doubt or denial of able men had caused to fall into oblivion.

"Dr. Gall having established, as it appears, from this progression of medullary bundles of the brain across the pons varolii, the thalami, and corpora striata, his law of the increase of the medullary fibres by the cineritious substance, wishes to apply it to the cerebellum.

"He has recourse here to the cineritious body of so singular a figure, which is found in the substance of the crura cerebelli, which has been called *corpus ciliare*, or *corpus fimbriatum;* the bundle called *processus cerebelli ad medullam* would give rise to the cerebellum, after having been reinforced by the corpus fimbriatum, as the pedunculi cerebri are by the thalami optici, and the cineritious part of the corpora striata. But perhaps this analogy is not complete. The corpus fimbriatum is enveloped, and, as it were, immersed in the medullary matter, instead of giving a passage to it; and we do not observe that it supplies it with any filament."

The fifth, sixth, and seventh articles must be considered together. They are all highly important towards a compleat understanding of the doctrine; the seventh particularly, expressing the possibility of unfolding the brain like a membrane, has been most talked of, but, like many other things, is least understood. To comprehend it we are to consider a garment stuffed or wadded, and afterwards convoluted and inclosed, so that if the inclosure is removed the contents may be unfolded into a smooth surface. On this subject the Reporters are by no means satisfied. The mode of proof they followed was equally candid and conclusive. Finding that the delicate texture of the brain would not admit a regular unfolding, they made incisions in such a manner as to perceive whether the blood vessels finished or ramified, each only in its separate folding; but neither this, nor any other mode of trial, was satisfactory to them. We shall transcribe the following passage to show the candour with which the whole Inquiry is conducted.

"These facts are accurate, but perhaps they only prove that there is less cohesion in the middle of a circumvolution, than in the rest of its capacity, and not that it is formed of two laminæ simply applied, and not adhering together.

"In other words, it may be admitted, in our opinion, that the white or intermediate portion of each circumvolution, is formed of two parts, which adhere to each other more slightly than the particles of each among themselves; or the union of them may be compared, for example, to that of the two laminæ of the dura mater, but not as was supposed, to that of the two sides of an intestine compressed, except, however, that the medium of union is not cellular matter, as in the dura mater; but the medullary substance itself a little softened.

"Besides, as this is a point of fact, admitting an appeal to the senses, we do not pretend to give to our opinion more authority than it ought to have; this question cannot be long of being examined mined by every anatomist, and will find as many judges as observers; it cannot fail then of being soon finally fixed.

"It is a great deal more difficult to demonstrate these orders of fibres in the cerebellum, than in the brain; and it is by analogy, rather than by actual knowledge, that Drs. Gall and Spurzheim admit their existence there.

"With regard to what they say of the commissures of the brain and cerebellum, their ideas are not new, or different from what has been already advanced by a very great number of anatomists; we may also add, that there is nothing in them which is not very probable."

The eighth and ninth article, relative principally to the office of the ganglions, do not differ materially from the opinions of many other anatomists. The tenth is in some measure conveyed in the fourth and some of the subsequent ones.

After a most candid examination, and a repetition of all the experiments, the Reporters conclude thus:

"Finally, it appears to us, 1st. That Drs. Gall and Spurzheim have the merit, not of having discovered, but of having recalled to the attention of physiologists, the continuity of the fibres which extend from the medulla oblongata into the hemispheres, and into the cerebellum, which Vieussens first detailed, and the decussation of the filaments of the pyramidal eminences described by Mistichelli, by Francis Petit, and by Santorini, but with regard to which, some doubt had been entertained.

"2d, That they are the first who have distinguished the two orders of fibres, of which the medullary matter of the hemispheres appears to be composed; the one of which diverges from the pedunculi, while the other converges towards the commissures.

"3d, That, by uniting their observations with those of their predecessors, they have made it very probable, that the nerves called cerebral ascend from the medulla oblongata, and do not descend from the brain; and that, in general, they have very much weakened, not to say overturned, the system which makes all the nerves come originally from the brain.

"But it also appears to us, 1st, That they have generalized rather in an inconsiderate manner, the resemblance of structure, and of functions in the various cineritious, or ash-coloured masses which are met with in the different parts of the nervous system.

"2d, That the idea which they entertain of a solution of continuity in the middle of the medullary matter of each circumvolution, which would permit of its being unfolded like a pipe or a purse, requires to be expressed in more definite terms than they have hitherto done, so as to indicate, that there is no complete proof of an absolute solution of continuity, but only of a more feeble cohesion.

"We ought however to remark, that these two articles do not affect their general result, with regard to the kind of separation and of reserve into which they placed the brain; and we ought, at the same same time to allow physiologists and pathologists to judge how far this kind of separation or laying aside, which anatomy seems to point out, is justified by facts, and how far it may favour the explanation of the numerous and astonishing phenomena of organic and animal life, and especially of those in which these seem sometimes dependant upon, sometimes distinct from, each other.

"The entering into all these questions would engage us in endless discussions, and foreign to our commission.

"Nor shall we propose to the class to decide upon the conclusion drawn by our anatomists, that there is no circumscribed place in the brain to which all sensations go, and from which all the voluntary motions issue, but that both of these functions may be exercised in a greater or a smaller extent of the nervous system.

"This opinion is undoubtedly that of Haller, Bonetus, and of the greater number of physiologists. There can be no doubt that it is from having confounded the metaphysical simplicity of the soul with the physical simplicity attributed to atoms, that the desire has originated of placing the seat of the soul in an atom; but the connection of the soul and body being necessarily incomprehensible to our minds, the more or less limited sphere which it is wished to give to the sensorium would not, in the smallest degree, assist our conception of it.

"But all these subjects are also too foreign to the business of the class; they are too slightly connected with physical facts; they lead to discussions too vague to form a proper object of attention for such a body as ours.

"We think ourselves, however, obliged to finish our investigation, by remarking, that, if we were even to adopt the greater number of the ideas of Drs. Gall and Spurzheim, we would still be far from knowing the relations, the uses, and the connexions, of all the parts of the brain.

"So long as we have not even a well-grounded conjecture with regard to the functions of the pituitary gland, of the infundibulum, of the mammillary eminences, of the cords passing from these eminences into the substance of the thalami, of the pineal gland, and of its pedunculi, we must apprehend, that any system whatever, with regard to the functions of the brain, is very incomplete, since it will not embrace these parts, so numerous, so considerable, and so intimately connected with the whole of this important organ.

"This is almost finishing with as much doubt, and as much uncertainty, as we began; but one can only require, on every subject, the degree of probability of which it admits; and the natural philosopher always performs his task very well, when he neither exaggerates nor diminishes this probability, and when he fixes its degree with precision.

'It is of importance to repeat once more, if it were only for the information of the public, that the anatomical questions with which we have been engaged in this report, have no immediate and necessary necessary connection with the physiological doctrine taught by Dr. Gall, with regard to the functions and the influence of the relative size of the different parts of the brain, and that all that we have examined with respect to the structure of the encephalon might be equally true or false, without any conclusion being drawn for or against this doctrine, which can only be determined by quite different means."

A sixteenth Number of the Enquirer, canvasses the following question. 'Does a minute knowledge of anatomy, contribute greatly to the discrimination and cure of diseases.' Of this paper, we shall transcribe the concluding paragraph.

"We cannot, therefore, but condemn the proposal of Dr. Beddoes, in directing the attention of the medical student, (we speak not of surgery) for so long a period as four years, to the cultivation of anatomy. Having endeavoured to shew how little the knowledge, that constitutes the physician, is connected with minute anatomical observation, and how absolutely and indispensibly necessary is the cultivation of *clinical* medicine, or the actual superintendence of the treatment of diseases, as the almost exclusive source of pathological and therapeutic science; we can only add, that a general knowledge of the structure of the animal body, especially of the structure, relative situation, and connection, of the viscera, appears to be all that is requisite to form the most able physician, even an Hippocrates or a Sydenham."

If Dr. Beddoes had proposed, that the student should devote four entire years to the study of *dissection* only, we should have been of the same opinion as Medicus. But when we speak of clinical medicine, we should hope to include the examination of those bodies, whose cases had been attended to during life; and a comparison with the appearance discovered in those who died of different diseases, or without any appearance of disease in those organs, the morbid effects of which are discovered in the first. Now for this purpose, anatomical knowledge cannot be too minute, especially for those practitioners who reside at a distance from the metropolis, or from those towns in which practical anatomy is taught.

---

### *Mr.* Wadd, *on Strictures in the Urethra.*

( Continued from our last. )

"It is not a little surprising, considering the respect so universally paid to Mr. Hunter, that his doctrines on this important subject, should have been so soon departed from by his immediate disciples. Mr. Hunter states three cases only, in which he considers the application of caustic to be justifiable : 'First, where the stricture is so tight as not to admit the smallest bougie to pass. Secondly, where the orifice in the stricture is not in a line with the urethra. Thirdly, where the passage has been obliterated by disease, and the urine passes by fistula in perinæo. The first very rarely occurs,' says Mr. H. 'for if the passage in the stricture be in a line with the general canal, a small bougie will commonly pass, and although it may not readily do so upon every trial, it will be sufficient to make way for another bougie, and THAT IS ALL THAT IS WANTED.' It is evident, Mr. Hunter considered the remedy in the other two situations, as only a forlorn hope, when he says, that in cases of this sort, he has succeeded beyond his expectation. To the ordinary cases of stricture, the application of this violent remedy, must of course be more objectionable. We are told, that some trifling strictures have been removed by two applications of it, and some even by one. But to such cases as these, could it be necessary? The impediment which usually constitutes a recent stricture, is the contraction of a slender piece of the inner membrane of the urethra, only a few lines in thickness, and which may be broken down by a slight mechanical force. That the armed bougie is not in the least adapted for removing an obstruction of any extent, must be equally obvious. It could only act upon the anterior part of the stricture, without a prospect of burning the whole extent of the contraction. 'Even could we imagine,' observes an ingenious author, 'that it had this power, our judgment and common sense would revolt at the doctrine of this being the proper plan to be pursued.' In all the other cases of obstruction, as tumours, enlargement of the corpus spongiosum, &c. it must be equally inefficacious and improper."

The following introduces the remarks of Mr. Whateley.

"In 1801, Mr. Whateley's book, to which I have so frequently referred, made its appearance, in which he proposed to obviate these inconveniences, by securing a small quantity of powdered lunar caustic, at the end of a bougie, by means of common glue, which, when sufficiently hardened, was covered with a thin coating of bees wax. The advantages attending the bougie, prepared in this manner, are thus described:—'In the first place, the bougie may be of any size; and may be readily passed into, or a little beyond, such strictures as are extremely narrow. Secondly, from the protection afforded by the wax coating, no part whatever of the caustic touches the sides of the urethra, in its passage to the stricture. Thirdly, a determinate quantity of caustic may be applied with certainty. Fourthly, the caustic cannot be separated from the bougie. Fifthly, the caustic may be made to act on the whole surface of the stricture at each application. Sixthly, where there are more strictures than one, the caustic may be directed and confined in its action. Seventhly, fixing the caustic with glue has this additional recommendation; we can attach it with safety to the very extremity of the bougie, and thereby apply it with more certainty to an impervious stricture, than is practicable with the common armed bougie.'

"However, these seven reasons, so systematically arranged, are insufficient to support the improved practice, more than three years. For in the year 1804, a more efficacious, less painful, and less hazardous remedy, is proposed. This remedy is the kali purum, or or pure potass, once called lapis infernalis; and which is in common use for making the deepest slough. At first sight it appears strange, that the most ungovernable of the whole fraternity of caustic substances, should turn out to be an 'easy, mild, efficacious remedy.' The reader, perhaps, may be a little inclined to scepticism on this point, particularly if he should happen to recollect, that Mr. Home holds nearly the same language of the lunar caustic. But we are informed, that the kali purum acts upon different principles. It is true, the lunar caustic takes off the cuticular lining of the urethra: not so the kali purum—that only *abrades*—and the degree of abrasion is, moreover, under the control of the operator, whose business seems to be, to effect a sort of chemical combination between the disease and the remedy. The mode of proceeding is as follows:—A bougie is chosen just large enough to enter the stricture with some degree of tightness; at the round end of which a small hole is to be made with a pin; into which a small portion of kali purum, less than half the size of the smallest pin's head, is to be placed; the hole is then to be contracted by the fingers, and the remaining vacancy filled up with hog's lard. The operator having oiled it, is to pass it by a gentle motion to the anterior part of the stricture, where it is to be kept for a few seconds, until the caustic begins to dissolve. It is then to be pushed forward about one eighth of an inch, and there retained a second or two, and then carried forward through the stricture. By this means, the kali being diffused over every part of the strictured surface, produces a slimy substance, compounded probably of the kali, the abraded matter of the stricture, and the oil and lard used in the operation. In this manner, says Mr. Whately, the kali penetrates, and dissolves the hard and diseased surface of a stricture, with a facility which no other remedy, that can be safely applied, will equally do. The safety, however, of this remedy, can only consist in the smallness of the portion of kali directed to be used; for it is hardly to be doubted, that if it be employed to have any efficacy as a caustic, it cannot be less injurious, or objectionable, than the lunar caustic."

Having thus stated the dangers and advantages of the different modes of applying caustic bougies, the author commences that part of his work, which, however old it may be, appears at present almost in a novel shape. The principal objections, it is observed, against the common bougie, are, that the cure is not so permanent as by the caustic, and that the irritable state of the urethra is kept up and increased by it. In answer to these, it is shown that the cures by the common bougie, though by no means permanent with any certainty, are as much so as those by the caustic, and that it can scarcely require any argument to prove that the irritability of the parts is likely to be much more increased in proportion to the severity of the remedy. Lest the proof of this proposition should rest merely on the author's assertion, he produces some authorities in confirmation of it, as well as cases which have occurred in his own practice, and insists with much propriety propriety on the rules laid down by Mr. Hunter, after a life spent in close investigation, assisted by comprehensive genius and extensive practice. But this opinion does not rest altogether on Mr. Hunter. The French surgeon, and among the English, the venerable names of Pott and Hawkins, as well as the more modern ones of Howard and Bell, all confirm the safety and advantage of the common bougie. Even Mr. Whately, it appears, speaks highly of the common bougie; produces several cases in which he succeeded with no other means, and considers it as unjustifiable to use any other, if we can succeed with this. It is not a little remarkable that one of his worst cases was, by no other remedy, relieved four times in seventeen years. From these, and a variety of other arguments and authorities, the author has perfectly satisfied us, that "the use of the common bougie should always be the leading principle in the cure of every description of stricture in the urethra." Several cases follow, in confirmation of the practice; selected as the most pointed from several which fell under his own observation, and in some of which he was engaged in consultation with gentlemen, the greatest advocates for the caustic.

Such are the contents of this performance, on a subject which for some time has been almost overlooked, but to which we are glad to see the public attention called by so judicious and well written a pamphlet.

---

*Observations on the Egyptian Ophthalmia, and Ophthalmia Purulenta, as it has appeared in England.* By WILLIAM THOMAS, Member of the Royal College of Surgeons, and Assistant Surgeon in the 11th Royal Veteran Battalion.

Mr. THOMAS is of opinion that the ophthalmia purulenta, at present so distressing in the British army, is the Egyptian ophthalmia, somewhat lessened in violence by transplantation to a Northern climate.

"*Specific Contagion* seems to be generally admitted as the cause of the *Ophthalmia Purulenta*, as it appeared in this country, and independent of those from which originally arose the true *Egyptian*, or *Ophthalmia Vera.* Of the specific properties of this contagion we must probably remain ignorant, as not being within the sphere of physical inquiry, or human investigation; but it may be presumed, that a disease so highly inflammatory as the Egyptian ophthalmia, in which the secretions are great, may be changed from it's simple state, by European habits, from their mode of living, by being mixed in numbers, and crowded into hospitals, after inflammation had taken place; causes which are at all times known to be favourable to propagating contagion, and probably did in this instance convert a simple inflammatory disease into a contagious one.

"The mode in which the *Ophthalmia Purulenta* has been propagated and communicated through the army in this country, is a sufficient proof of the high degree of contagion it suddenly acquires, after it has apparently vanished, and for weeks and months lain dormant, and long after the local causes from which it arose may be supposed to have ceased to act: removal from the country, though it may supend for a time it's influence, does not put a stop to it altogether, nor dispossess it of the power of being communicated to corps that never have been exposed to the influence of an Egyptian climate. The manner in which it is supposed to be most commonly communicated, would induce one to believe that it bears a very strong affinity with other infectious diseases transferred by local contact; but there is reason to suppose the *Ophthalmia Purulenta* is at times spread by more general means, as by the bedding and by the atmosphere of a barrack-room, where the disease has prevailed. Whether the mode by which it is conveyed may make any difference in its nature or violence, may be difficult, if not impossible to determine; but it is certain, that which ever way the disease is caught, it always bears the same characteristic marks which distinguish it from other inflammations of the eye; yet may vary astonishingly in the degrees of inflammation, the irritation it produces, the alteration in the parts concerned, and the quantity of purulent discharge."

The author suggests a doubt, whether the disease may not be milder when taken by effluvia, than when the matter is immediately applied to the part; but this is not confirmed by any certain experiment. As an early mark of the disease, we are advised to examine the appearance of the caruncula lachrymalis, which we are informed is larger and fuller than usual, some days before any of the acute symptoms come on. Other appearances are particularized, all of which it is absolutely necessary to detect as early as possible, the whole advantage of treatment depending on the early application of remedies.

It would seem by Mr. Thomas's account, that the Military Ophthalmia had lost much of its severity, since in the numerous cases here described, that bold venesection, hitherto so much recommended, was found unnecessary. The principal remedy recommended by the author, was an ointment composed of kali purum, and the spermaceti ointment in the proportion of ℈j. to ℥ij. Before this was applied, the eyes had been frequently subjected to cold watery affusion, and were carefully cleansed; so that the ointment might without difficulty touch every part.

Some remarks on the early Egyptian writers, show the learning and industry of the author, but do not add greatly to the value of the work.

*A Letter to the Honourable and Right Reverend the* LORD BISHOP OF DURHAM, *President; and the other Members of the General Committee of the Society for bettering the Condition of the Poor, Proposing a Plan for Improving Dispensaries, and the Medical Treatment of the Diseased Poor.* By JOHN HERDMAN, M. D. Member of the Royal College of Physicians London; of the Medical Society, Edinburgh; and one of the Physicians of the City Dispensary, &c.

THIS little tract commences with a brief account of the various sources of diseases, preparatory to the methods of cure. Under the latter, the author very judiciously reminds us, that we are to consider dietetics as well as remedies.

"Now, in this (observes Dr. H.) lies the imperfection, or inadequacy of Dispensaries; they only afford the medicinal part of the treatment of diseases. The dietetic part, the other instrument of the physician's power, he cannot command. But the necessity and utility of this powerful auxiliary are unquestionable. The deplorable condition of many of the suffering objects, who make application at Dispensaries, shew it beyond all doubt or dispute.

"Disease and indigence are the leading features of their character. Without all the comforts, and possessed of only a scanty share of the common necessaries of life, their health at best stands on a very slender basis. Thus, they are predisposed to diseases, while they are exposed to a variety of their immediate causes, and therefore they suffer a number of both general and local maladies.

"The labouring people are certainly exposed to many casualties from which the higher orders of society are shielded:—to damps and cold contracted by working in wet weather; by the want of a change of raiment, deficient bed cloths, cold rooms and cottages; hurts, wounds, and other accidents peculiar to their situation as out-door labourers; and, therefore, they require medical and surgical assistance more frequently than others, whose occupations expose them less to the inclemency of the weather. In disposing, therefore, of their labour, (their only stock in trade) they are not on a footing with other classes of the community, since they are liable to contract various diseases, and often suffer much from want of proper medical assistance, and other necessary comforts, which those of a higher rank enjoy. Nothing can exceed, on many occasions, the sufferings of this useful class, upon which the strength, stamina, and riches of the country depend."

Some very judicious remarks follow on the important scale which the labouring class hold in society. To preserve them from the consequences of all the distresses attending sickness united with poverty, it is proposed that Dispensaries should be so constructed, that relief may be afforded not only in medicine, but in those articles of diet and warm covering, which are procured with more difficulty in proportion as they are more necessary.

"But

"But in order (continues our author) to give efficiency to this branch of the healing art, among the poor of this extensive metropolis, it is humbly proposed:—

"1st. That the Cities of London and Westminster, with their environs, should be divided into certain districts, and that each district should have a Dispensary, centrally situated, and provided with all the powers of the healing Art.

"2dly. That the affairs of the Dispensary should be managed by a Committee of its Governors, or those who contribute towards its support, including the Clergymen, Church-wardens, and Overseers of the parishes within its boundaries.

"3dly. That each district should support its own Dispensary, by voluntary contributions, &c.

"4thly. That a Fund should be established, under the management of the Committee, for receiving the deposits of those industrious and virtuous poor, who might be disposed in the day of health to lay up something for the day of disease.

"5thly. That the Medical Gentlemen of the Dispensary should have the same command of the dietetic, as they now have of the common medicines, and thus be able to bring the whole powers of the healing Art to bear in the treatment of the diseased poor.

"6thly. That a certain number of the Committee should be selected, or formed into a Sub-Committee, including a Church-warden and Overseer of each parish within the district, to assist the Medical gentlemen on particular occasions, in enquiring into the worldly condition of the patients."

It is scarcely doing justice to such a work, briefly to announce its contents. Many parts of the plan, which may at first appear impracticable, are shown not to be beyond the reasonable expectation of those who embark in earnest in such a cause. But whatever difficulties may occur, we cannot sufficiently applaud the humanity of the author in the industry he has shown, nor his prudence in the society, whose attention he has called upon, for the completion of such an object.

---

*New Medical Compendium, for the Use of Families, &c. considerably enlarged and improved.* By D. Cox, Chemist to his Majesty. Glocester, 1808. pp. 416.

The author's principal object in this publication, is to recommend and explain the use of his family medicine chests. Such a stock of valuable medicines, with such plain directions for their use, cannot fail to be of great service to persons residing at a distance from large towns; the distance alone too frequently renders it impossible to procure proper medical advice in time to save many patients, attacked by acute diseases. The directions and advice here given, appear to be judicious and correct. A number of valuable observations on culinary processes and the preparation of bread are interspersed thro' the work, which much enhance its value.—It is not, however, to be supposed, that this or any other work of an individual, dual, can be put in competition with the Pharmacopœas of the Royal Colleges; or that we should recommend it to the students of medicine, who must draw their information from the highest sources.

The author concludes his work with a very useful glossary of medical and chemical terms, and a copious index.

---

*A Syllabus of a Course of Lectures on the Elements of the Medical Sciences, and on the Practice of Physic. To be delivered at the Middlesex Hospital.* By Thomas Young, M. D. F. R. S. &c. London, 1809.

Our reason for noticing a local publication of this kind, is to shew our readers how great a revolution in Chemical Theory and Nomenclature has been produced by Davy's discoveries. Dr. Y. divides chemical substances into eight Classes, viz. Elements, Alkalies, Oxids, Compositions, Acids, Semi-acids, Salts, and Semi-salts. The first class or elements, he divides into two orders. The *first* order he denominates *Empyreal*, which contains only one genus, viz. *Oxygen*. The *second* order is called *Metalic*, and contains forty-two genera, viz. Hydrogen, Nitrogen, Potassium, Sodium, Baritium, Tellurium, Uranium, Antimony, Manganese, Arsenic, Zinc, Tin, &c. &c. through all the metals. The *third* order of elements is termed *Pyrophorie*, and contains three genera, viz. Carbon, Phosphorus, and Sulphur. The *fourth* order is denominated *indeterminate*, and contains three genera, viz. Muriatium, Fluatium, and Bocacium. Class II. Alkalies. 1. *Simple*, Potass, Soda, Barita, Strontia, Lime, Magnesia. 2. *Compound*, Ammonia. Class III. Oxids. 1. *Simple*, Alumina, Glycina, Itria, Zirconia, Silica. 2. *Binary* oxids, Hydrastagnal gas, Stagnal gas, Ether, Alcohol, Camphor, Essential oil, Resin, Wax, &c. &c. This specimen is sufficient to inform our readers of the important changes produced by the new discoveries of Mr. Davy, which is all we proposed.

